# Nanosensor for the detection of bromothymol blue dye and its removal from wastewater by sustainable methods†

**DOI:** 10.1039/d4ra08296f

**Published:** 2025-02-07

**Authors:** Nashra Sheraz, Afzal Shah, Syed Sakhawat Shah

**Affiliations:** a Department of Chemistry Quaid-i-Azam University Islamabad 45320 Pakistan afzals_qau@yahoo.com

## Abstract

This study introduces the environmentally friendly synthesis of Ag_2_O, TiO_2_, and Ni-doped SnO_2_ nanoparticles (NPs) and their application in detecting and removing bromothymol blue (BTB) dye from wastewater. The unique electronic properties and quantum size effects of NPs allow them to surpass conventional materials. Characterization of the synthesized nanoparticles was conducted through spectroscopic and voltammetric techniques. TiO_2_ NPs, in conjunction with amine-functionalized multiwalled carbon nanotubes (NH_2_-fMWCNTs) enhanced the sensitivity of the transducer, while electrochemical impedance spectroscopy confirmed effective charge transport through the designed sensing platform. The sensor was found to exhibit the qualities of repeatability, specificity, and reproducibility, achieving a detection limit of 0.1 nM for BTB dye. For wastewater purification from BTB, Ag_2_O NPs were employed as a photocatalyst and the photocatalytic degradation monitored with electronic absorption spectroscopy revealed a 92% degradation of BTB dye within 30 minutes. Furthermore, Ni-doped SnO_2_ NPs were utilized for the adsorptive removal of the dye, demonstrating a maximum adsorption capacity of 90.90 mg g^−1^. The adsorption mechanism adhered to the Langmuir model at lower BTB concentrations and the Freundlich model at higher concentrations, with kinetics aligning with the intra-particle diffusion model. This research underscores the promise of electrocatalytic and photocatalytic nanomaterials as scalable, sustainable, and eco-friendly approaches to combat water pollution.

## Abstract 2

This study introduces the environmentally friendly synthesis of Ag_2_O, TiO_2_, and Ni-doped SnO_2_ nanoparticles and their application in detecting and removing bromothymol blue dye from wastewater.

## Graphical abstract

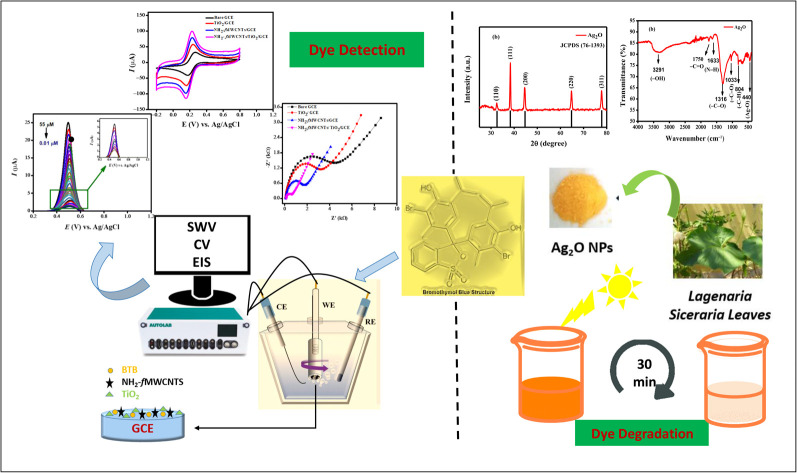

## 1 Introduction

Water, an essential resource for sustaining life, is increasingly at risk from pollutants, particularly as industrial activities continue to advance and expand. The population explosion and rapid industrial advancements to improve living standards cause serious environmental problems, especially in underdeveloped countries.^1^ Industrial revolution has brought luxury to life by transforming agrarian society to industrial society. However, industries have also caused environmental damage. The unregulated discharge of polluted industrial wastewater has significantly harmed the ecosystem. The textile industry is regarded as a major polluter which utilizes more than eight thousand chemicals and consumes an enormous amount of water. Tainted water sources, heavy industrial emissions, unsanitary conditions and improper waste disposal associated with these industries release toxic effluents into water reservoirs which change the pH, color, density and taste of water. These industries discharge different kinds of pollutants such as dyes, heavy metals, salts, emulsifiers, leveling agents, *etc.* which contaminate water bodies. These contaminants make water unsafe and unusable for humans and disrupt aquatic life.^2,3^

Bromothymol blue (BTB) is a derivative of textile dyes that belongs to the family of sulfonephthalein. It is a halochromic material that appears yellow in acidic media, green in neutral media, and blue in basic media.^4,5^ It is widely used as an acid–base indicator and biological stain. It also finds its application in modern sensors, paper, textiles, medicine and food industries.^6^ Despite of their usage, BTB is also associated with harmful health hazards such as irritation of the eyes, skin, and respiratory tract. Intake of a high amount of BTB dye leads to vomiting, nausea, and even death.^7^ The discharge of BTB into aquatic environments can lead to considerable disturbances within these ecosystems. Its intense coloration diminishes light penetration, adversely affecting photosynthetic organisms like algae and aquatic plants, thereby disrupting the entire food chain. Additionally, as a pH-sensitive dye, BTB can modify the water's pH, posing risks to organisms that are vulnerable to such changes, which raises concerns regarding water quality and highlights the need for research on pH-altering pollutants. BTB's stability and resistance to natural degradation render it a persistent contaminant, emphasizing the necessity for effective removal strategies and its significance in wastewater remediation studies. Serving as a model for examining the environmental effects of similar dye pollutants, BTB is widely utilized across various sectors, including laboratories for pH indication, medical diagnostics, and biological research. Its prevalent use increases the probability of its occurrence in wastewater, making it a prime candidate for research focused on developing innovative detection and remediation technologies.^5,8,9^

Our goal is to create an economical strategy for identifying and eliminating BTB dye from wastewater. We intend to attain accurate detection *via* electrochemical techniques, specifically by employing advanced materials to improve sensitivity and selectivity. For the removal process, we plan to investigate effective adsorption methods and photocatalytic degradation utilizing green-synthesized materials, which offer sustainable and cost-effective solutions for dye remediation. This strategy promotes environmental sustainability by reducing chemical waste and energy usage in water purification efforts.

Among different analytical techniques for the detection of pollutants, special attention has been paid to electro-analytical techniques owing to their excellent sensitivity, selectivity and reproducibility. The designing of electrochemical sensors demand suitable electrode modifiers that could act as a bridge between the transducer and the analyte molecules.^10^ Chemically modified electrodes (CMEs) are attracting attention in sensor applications due to their ability of eliminating the problems *i.e.* high redox potential and low limit of detection (LOD) and limit of quantification (LOQ) associated with unmodified electrodes. Appreciable mechanical resistance, chemical inertness, high conductivity, and large surface area of carbon nanotubes (CNTs) have great significance for their use in sensor designing.^11^ Functionalization of CNTs with amino or carboxyl groups increases their application horizon. Functionalized MWCNTs combine through the Π–Π stacking to the organic compounds results in good electrical conductivity. Metal nanoparticles are also employed as electrode modifiers to amplify the selectivity, stability and overall performance of the designed sensors. As they possess a high surface-to-volume ratio, the analyte can interact with more active sites, thus, resulting in signal intensification of the analyte of interest.^12,13^ The combination of carbon-based nanomaterials and metal nanoparticles are used as electrode modifiers in sensor design to get the best results. Based on these considerations NH_2_-fMWCNTs and TiO_2_ NPs were used in the present work for electrode modification.

As freshwater sources are rapidly depleted, therefore the development of an efficient and sustainable technique gains the limelight in order to reuse dye-containing wastewater. Although there are a variety of methods reported so far for the removal of dyes, however, most of them are inappropriate for practical applications owing to long sequence of operations, high cost, and production of secondary pollutants. Efficient and ecologically friendly dye removal is achieved by adsorption and photocatalytic methods.^14^ Nanotechnology can tackle the issue of wastewater purification through the development of materials possessing unusual properties. Fabrication of different nanoparticles having unique morphologies and properties has grasped the attention in today's world.

In recent years, metal oxide-based photocatalysts have shown wide potential for the purification of wastewater. Semiconductors with narrow band gaps, such as silver oxide, which has a band gap of 1.2 eV, are capable of absorbing a significant portion of the solar spectrum, thereby enhancing photocatalytic activity.^15^*Lagenaria siceraria*, commonly known as bottle gourd, presents considerable promise for the environmentally friendly synthesis of nanoparticles, especially silver oxide nanoparticles. The presence of bioactive phytochemicals in bottle gourd, such as flavonoids, saponins, triterpenoids, alkaloids, and phenolics, aids in the reduction of metal salts and the stabilization of nanoparticles. These phytochemically synthesized silver oxide nanoparticles have potential applications in the degradation of pollutants in both water and soil. The stabilizing phytochemicals may enhance the reactivity and stability of the nanoparticles, making them effective catalysts for the breakdown of dyes and other organic contaminants in wastewater treatment. Furthermore, utilizing *L. siceraria* for nanoparticle synthesis represents a sustainable method that avoids the use of harmful reducing agents and solvents, thereby minimizing environmental impact. This green synthesis approach is also cost-effective, relying on readily available plant materials instead of costly and hazardous chemicals, and is typically performed at room temperature, which reduces energy consumption and further contributes to its environmental advantages.^16,17^

Similarly, the mechanical and chemical stability of SnO_2_ makes them suitable for various applications such as sensing, adsorption, light energy conversion, *etc.* Doping of metal oxides especially with magnetic elements further widens their application horizon. It has been reported that nickel is one of the transition metals that enhances the properties of SnO_2_.^18^ The motivation of this study was to detect BTB dye through an efficient sensing scaffold and their removal through cost-effective and environmentally benign methods.

This report represents, to our knowledge, the first comprehensive study focusing on both the detection and removal of BTB dye. The detection goal is accomplished through a highly sensitive electrochemical platform, while the removal aspect is facilitated by Ag_2_O and SnO_2_ NPs. The current work presents an innovative integration of advanced materials and green chemistry principles that greatly improve environmental remediation initiatives. Our analytical detection technique is exceptionally sensitive and eco-friendly. The creation of a sensor that integrates NH_2_-fMWCNTs with TiO_2_ for the detection of BTB dye exemplifies an advanced innovative strategy in electrochemical sensing, capitalizing on the conductive attributes of carbon nanotubes and the catalytic properties of titanium dioxide. This integration significantly improves the sensor's sensitivity and selectivity, as the NH_2_-fMWCNTs promote efficient electron transfer while TiO_2_ enhances surface reactivity at the transducer surface by providing more sites for preconcentrating BTB molecules. Employing Square Wave Voltammetry (SWV) as the detection method facilitates high-resolution analysis with swift response times, allowing for accurate detection of BTB dye even at low concentrations. This novel configuration not only expands the possibilities for identifying organic dyes in environmental samples but also enhances the functionality of SWV-based electrochemical sensors for detecting pollutants in complex media containing interfering species. The nanoparticles, produced through environmentally friendly methods, demonstrate robust catalytic capabilities that enable rapid and effective pollutants degradation. The green synthesis approach not only reduces the environmental impact of the nanoparticle production process while improving their stability and biocompatibility, rendering them suitable for applications in sensitive ecological contexts. Our synthesized photocatalyst and designed dye degradation method demonstrate efficient photocatalytic degradation of BTB dye, achieving a remarkable 92% breakdown within 30 minutes. There is no existing literature that reports the degradation of BTB dye in such a shorter duration. The effective degradation capabilities of Ag_2_O nanoparticles highlight their promise as a sustainable approach for contaminant treatment, providing a swift, economical, and environmentally friendly alternative to traditional degradation techniques in environmental remediation initiatives.

Additionally, nickel-doped SnO_2_ nanoparticles were used for the first time as an adsorbent for BTB dye, exhibiting an adsorptive removal efficiency of 91.7%. The incorporation of nickel enhances the surface activity and adsorption capacity of tin oxide, allowing for more efficient binding of dye molecules. This modification not only increases the overall effectiveness of dye removal but also improves the stability and reusability of the adsorbent. Advancements in nanoparticle-based adsorption present a novel and sustainable solution for addressing organic dye contamination in water treatment. However, the transition from laboratory-scale to large-scale applications necessitates a thorough assessment of upscaling and economic viability. It is crucial for industrial production to focus on cost-effective and environmentally friendly methods, such as green synthesis, to ensure sustainability while upholding quality standards. Important considerations include the costs of raw materials, energy consumption, operational expenses, and the management of nanoparticle recovery or disposal to mitigate secondary pollution. Additionally, it is imperative to tackle challenges related to stability, agglomeration, and system compatibility. Conducting small-scale preliminary experiments and cost analyses is essential for overcoming these obstacles, thereby facilitating efficient and economically feasible wastewater purification.

## 2 Experimental

### 2.1 Instruments

Voltammetric measurements were conducted using the Metrohm Multi Autolab Cabinet Model No. MAC80146 from Utrecht, The Netherlands. EIS was performed using the Gamry Interface 5000E Potentiostat. Structural analysis was performed with a Phillips Xpert Pro 3040/60 X-ray diffractometer. The Shimadzu UV-Vis spectrophotometer model 1700 facilitated the monitoring of BTB dye adsorption and its degradation under solar light. FTIR spectra of the synthesized nanoparticles were recorded using a Shimadzu 8400S FTIR spectrometer.

### 2.2 Synthesis scheme

#### 2.2.1 Synthesis of TiO_2_ NPs

**Scheme 1:**
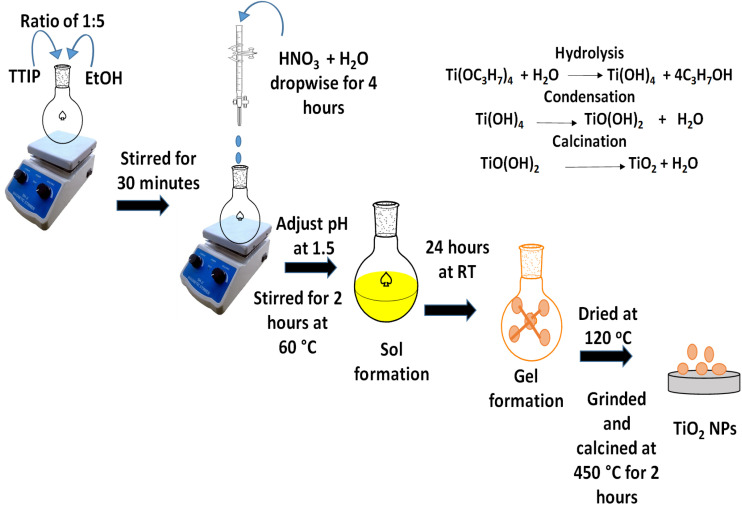
Sol–gel method for the synthesis of TiO_2_ NPs.

TiO_2_ nanoparticles were synthesized using a modified sol–gel method as illustrated in Scheme 1.^19^ Tetra titanium isopropoxide (TTIP) served as the precursor, while ethanol acted as the solvent. A mixture of TTIP and ethanol in a 1 : 5 ratio was prepared in a flask and stirred for 30 minutes. Subsequently, a solution of nitric acid and water was added dropwise over a period of four hours until a pH of 1.5 was achieved, followed by an additional two hours of stirring at 60 °C to facilitate complete hydrolysis. Once the sol was formed, it was stirred for another 24 hours at room temperature to promote gelation. The resulting gel was dried at 120 °C to eliminate organic solvents, then ground and calcined at 450 °C for two hours, yielding white powder TiO_2_ nanoparticles.

#### 2.2.2 Synthesis of Ag_2_O NPs

**Scheme 2:**
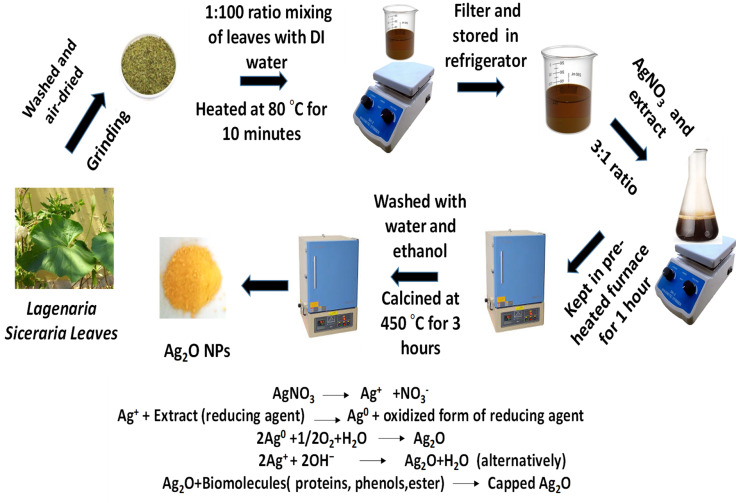
Synthesis scheme of Ag_2_O NPs.

Ag_2_O NPs were synthesized using an environmentally friendly method involving the extract of *Lagenaria siceraria* leaves, as illustrated in Scheme 2.^20^*Lagenaria siceraria*, commonly contains bioactive phytochemicals such as flavonoids, saponins, triterpenoids, alkaloids, and phenolics, proteins which helps in the reduction of metal salts and the stabilization of nanoparticles. The process began with the collection of *Lagenaria siceraria* leaves, which were thoroughly washed four times with distilled water to eliminate any dust and contaminants. The leaves were then air-dried in the shade for five to six days. Once dried, they were ground into a fine powder. Subsequently, five grams of this powder were mixed with 500 mL of distilled water and stirred for two hours. The extract was then filtered and centrifuged to remove any dissolved impurities, and it was stored at a temperature of 3–4 °C for later use.

A 1 mM silver nitrate solution was mixed with the previously prepared extract in a flask at a ratio of 3 : 1 and stirred at room temperature for 10 minutes to ensure complete homogenization of the two solutions. Subsequently, the flask was transferred to a pre-heated furnace set at 100 °C for one hour to enhance the reaction rate by reducing its activation energy. Following the precipitation process, the mixture was washed three times with water and ethanol, then calcined at 450 °C for three hours. The resulting capped nanoparticles were milled and stored in a sealed container.^21^ The primary capping agents consist of phenols, proteins, and esters. The FTIR spectrum of Ag_2_O nanoparticles displays peaks that correspond to the functional groups such as (C–O) and (N–H, C=O) present in capping agents.

#### 2.2.3 Synthesis of nickel doped SnO_2_ NPs

**Scheme 3:**
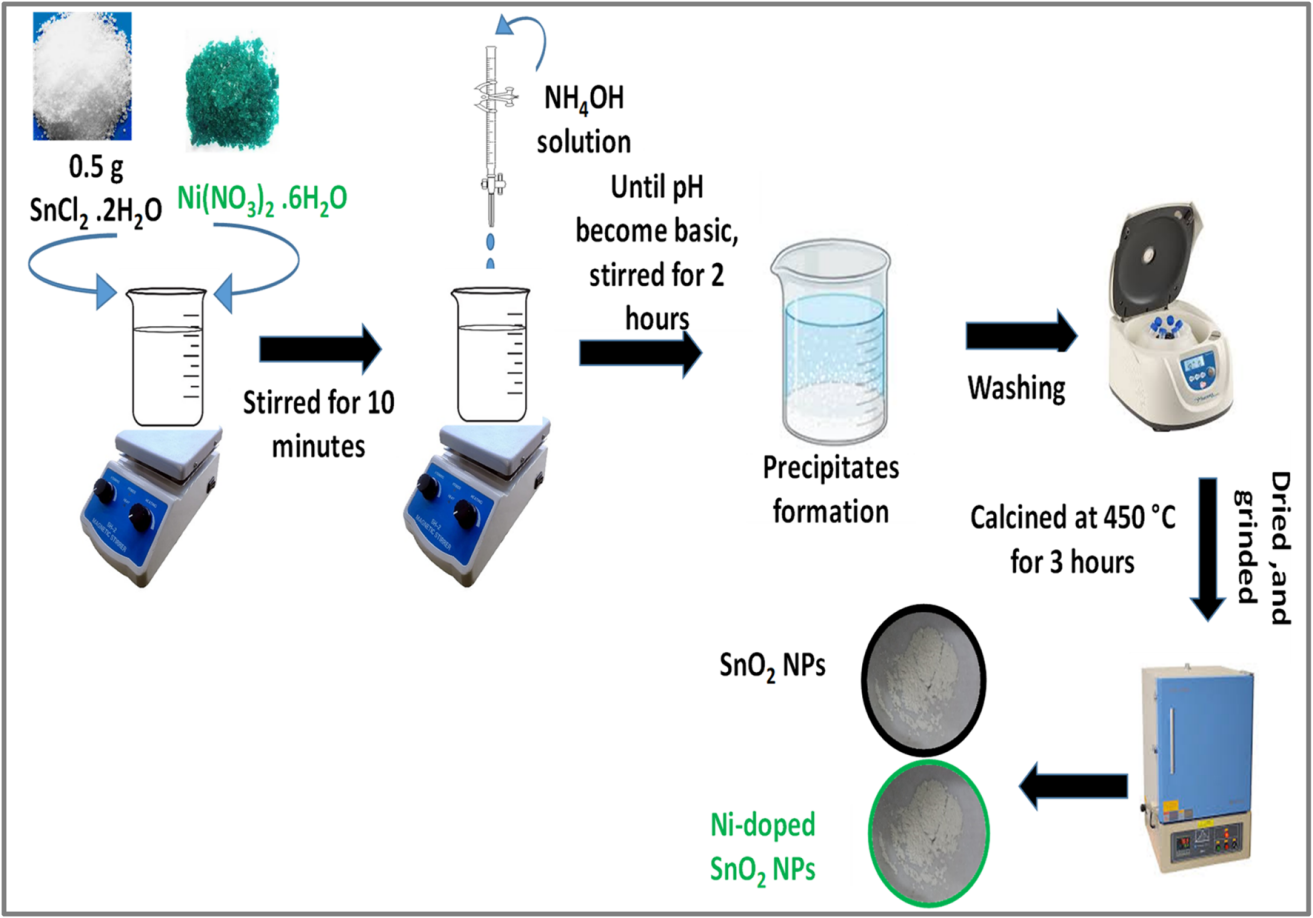
Protocol for the synthesis of doped and undoped SnO_2_ NPs using coprecipitation method.

Ni-doped SnO_2_ nanoparticles were synthesized by co-precipitation method.^22^ Initially, 0.5 grams of tin chloride dihydrate was dissolved in 50 milliliters of distilled water and stirred for 10 minutes. Once the precursors were fully dissolved, ammonia solution was added dropwise until a basic pH was achieved, followed by stirring for two hours. The resulting mixture was allowed to settle, after which the precipitates were washed, ground, and calcined at 450 °C for three hours, resulting in the formation of tin oxide nanoparticles. For the Ni-doped SnO_2_ nanoparticles, the procedure remained consistent with that of SnO_2_, with the exception of incorporating a predetermined amount of nickel nitrate hexahydrate as a dopant prior to the addition of the ammonia solution.^22^Scheme 3 illustrates the synthesis process for both doped and undoped SnO_2_ nanoparticles.

### 2.3 Transducer modification method

Prior to each electrochemical test, the surface of the glassy carbon electrode (GCE) was polished with alumina slurry applied to a nylon rubber mat. Any particles produced during this process were eliminated by ultrasonically cleaning the transducer in a mixture of ethanol and water. To modify the cleaned GCE surface, slurry consisting of 1 mg per 1 mL of NH_2_-fMWCNTs and TiO_2_ nanoparticles, prepared in DMF, was applied using a layer-by-layer deposition technique. Following this, BTB dye was drop-casted onto the modified GCE surface and allowed to air dry. The resulting working electrode, designated as GCE/NH_2_-fMWCNTs/TiO_2_/BTB, was subjected to voltammetric analysis in an electrochemical cell containing 20 mL of supporting electrolyte solution.^23^ Pt wire and Ag/AgCl were used as counter and reference electrodes. Square wave voltammetry (SWV) was utilized to detect the BTB dye on the sensor's surface and to compare its signal with that from the unmodified GCE. Additionally, electrochemical impedance spectroscopy (EIS) and cyclic voltammetry (CV) were performed to assess the charge conduction properties of the sensor and to ascertain the electroactive surface area of the modified electrode.

### 2.4 Dye removal procedure

UV-Vis spectroscopy was used to investigate the photocatalytic degradation and adsorptive removal of BTB dye from wastewater. A BTB dye solution with a concentration of 55 μM was prepared by mixing 5% ethanol with 95% water, resulting in an absorbance close to 1.0. For the photocatalytic degradation, an appropriate amount of the photocatalyst was added to a 20 mL BTB dye solution, which was then exposed to sunlight while being continuously stirred magnetically. Samples of 2 mL were taken every 5 minutes using a micropipette, and the degradation was monitored *via* UV-Vis spectroscopy. Various factors, such as catalyst dosage, pH, and reaction time, were optimized to maximize degradation efficiency. To prevent photodegradation that could be induced by light exposure, the reaction mixture for the adsorptive removal of BTB dye was shielded with aluminum foil. The adsorption process was conducted under optimized conditions, which included an adsorbent concentration of 4 mg, a dye volume of 20 mL, a pH of 3, and a contact time of 20 hours, with UV-Vis spectra collected at regular intervals.

## 3 Results and discussion

### 3.1 Characterization

The materials synthesized for detection, photocatalytic degradation, and adsorptive removal were characterized using XRD and FTIR spectroscopy.

#### 3.1.1 XRD analysis

**Fig. 1:**
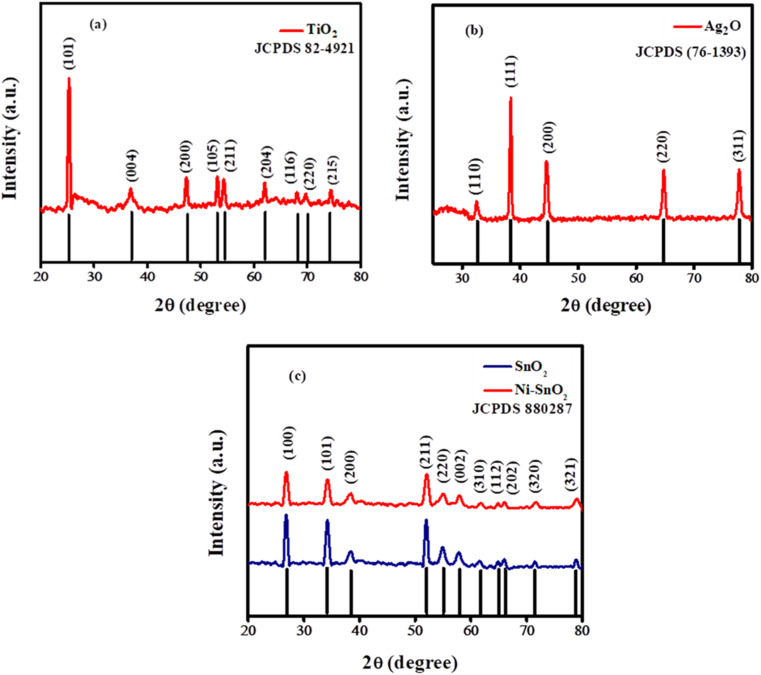
X-ray diffractograms of (a) TiO_2_ NPs (b) Ag_2_O NPs and (c) both doped and undoped SnO_2_ NPs.

X-ray diffraction analysis was conducted to assess the structural properties of the synthesized nanoparticles. Fig. 1(a) displays the XRD spectrum of the synthesized TiO_2_ nanoparticles, where the presence of sharp peaks signifies their crystallinity. The specific crystallographic planes were determined by comparing the peak positions with the standard JCPDS card. The identified planes, including (101), (004), (200), (105), (211), (204), (116), (220), and (215), align with the JCPDS database (89-4921), thereby confirming the formation of TiO_2_ nanoparticles. Additionally, the lack of extraneous peaks and the alignment of the observed peaks with the standard further validate the anatase phase of the TiO_2_ nanoparticles.^24^ Similarly, the XRD spectra for the synthesized Ag_2_O NPs, depicted in Fig. 1(b), reveal peaks corresponding to the crystallographic planes (110), (111), (200), (220), and (311), which align with JCPDS card number (76-1393). The sharp diffraction peaks at specific angles indicate the crystalline nature, purity, and cubic phase structure of the synthesized Ag_2_O NPs.^20,25^

The XRD spectra for both doped and undoped SnO_2_ nanoparticles are illustrated in Fig. 1(c). The recorded diffractograms clearly show that all detected peaks align with the crystallographic planes (100), (101), (200), (211), (220), (002), (310), (112), (202), (320), and (321), which correspond to the standard JCPDS (880287) indicating a tetragonal structure. Upon nickel doping, a slight shift of the peaks towards higher angles was noted, along with a decrease in peak intensity and an increase in peak width, suggesting successful incorporation of the dopant. The absence of secondary peaks in the diffractograms indicates that the crystalline structure is maintained, likely due to the comparable sizes of the dopant and host atoms.^26^ The synthesized materials displayed average grain sizes of 14 nm for TiO_2_ and 17 nm for Ag_2_O, as calculated using Debye–Scherrer's formula. The introduction of nickel led to a reduction in the crystallite size of SnO_2_ nanoparticles from 15 nm to 11 nm, indicating that nickel's presence hinders the growth of particle size.

#### 3.1.2 FTIR analysis

**Fig. 2:**
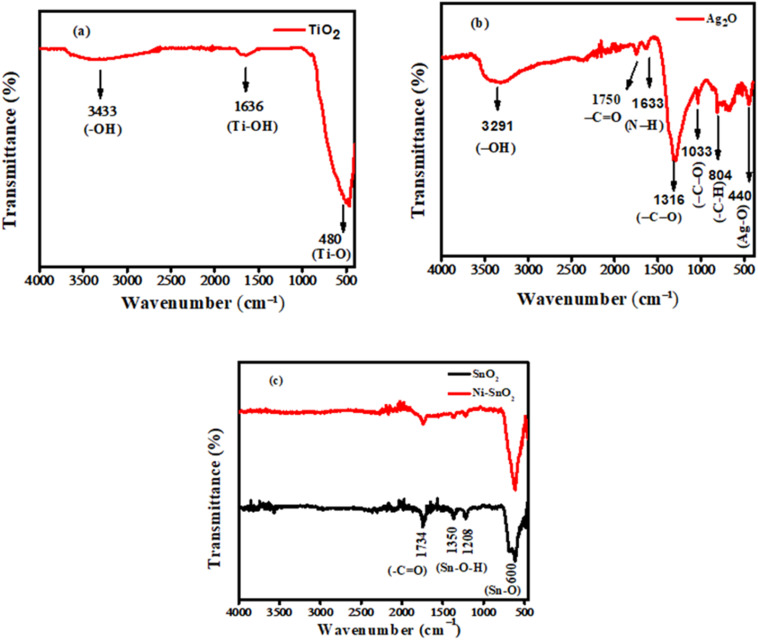
The FTIR spectra of the synthesized (a) TiO_2_ NPs (b) Ag_2_O NPs, and (c) SnO_2_ along with Ni-doped SnO_2_ NPs.

The infrared spectrum of the synthesized TiO_2_ sample is depicted in Fig. 2(a). A slight broad peak at 3433 cm^−1^ is linked to the stretching vibrations of hydroxyl groups (–OH), suggesting the existence of surface water adsorbed from the surrounding environment. Furthermore, the peak at 1636 cm^−1^ is related to the bending vibrations of hydroxyl groups in molecular water. The strong absorption band at 480 cm^−1^ corresponds to the distinctive vibrational mode of the Ti–O bond.^27^ As the peaks at 3433 and 1636 cm^−1^ are not that much significant so we can say that the synthesized sample is pure.

The FTIR spectrum of the synthesized Ag_2_O nanoparticles is displayed in Fig. 2(b). The spectral signal observed at 3291 cm^−1^ is associated with the O–H stretching vibrations of water molecules that may have been adsorbed due to environmental exposure. The peak at 1750 cm^−1^ is associated with the stretching vibration of the carbonyl group in amides, signifying protein presence. This is further supported by the appearance of N–H vibration at 1633 cm^−1^. The 1316 and 1033 cm^−1^ peaks correspond to C–O stretching of the phenolic and ester groups present in the extract. The peak observed at 804 cm^−1^ is associated with the bending vibration of aromatic C–H bonds. The signal at 440 cm^−1^ corresponding to Ag–O stretching vibrations confirms the formation of Ag_2_O NPs.^20^Fig. 2(c) presents the FTIR spectra of undoped and doped SnO_2_ NPs. The peak at 1734 cm^−1^ indicates the stretching vibration of the C=O bond. The signals appearing at 1350 and 1208 cm^−1^ are associated with the symmetric and asymmetric vibration of Sn–O–H bonds which indicates the presence of water molecules adsorbed on NPs. The peak appearing at 600 cm^−1^ is associated with the stretching vibration of Sn–O bonds. After doping the characteristic peaks of Sn–O slightly shift towards higher wavenumber which ensures the doping with nickel atoms.^28^

### 3.2 Electrochemical characterization

**Fig. 3:**
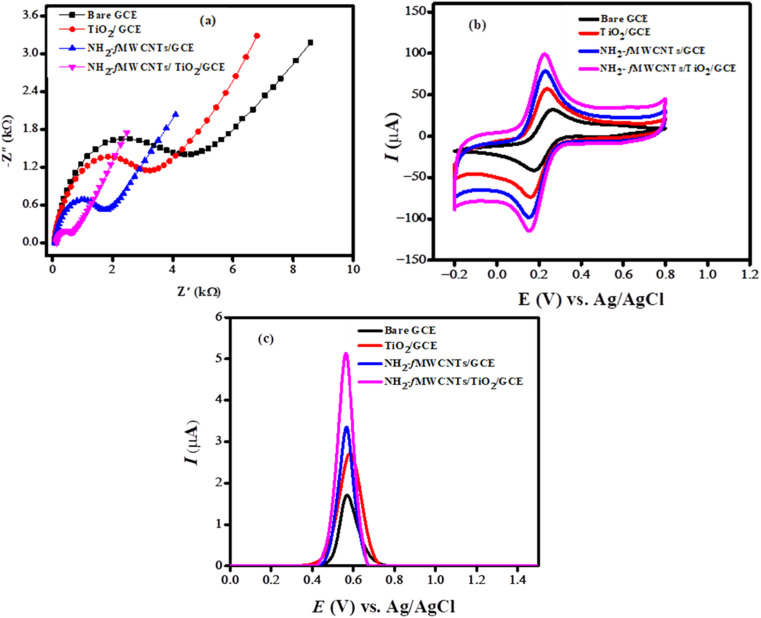
(a) Electrochemical impedance spectra presented as Nyquist plots illustrate charge transport characteristics across bare and modified GCEs; (b) CV responses of the redox probe at the bare and modified GCEs; (c) square wave voltammograms of 10 μM bromothymol blue recorded at the bare and modified GCEs in PBS of pH 6.0 at a scan rate of 100 mV s^−1^.

EIS gives information about the charge transfer properties of the electrochemical sensing platform. Various modified electrodes were subjected to EIS analysis for the evaluation of interfacial properties of the electrode–electrolyte interface. The semicircular section in the higher frequency area of the Nyquist plot illustrates the charge transfer resistance (*R*_ct_), which is determined by the diameter of this semicircle, whereas the linear section indicates processes limited by diffusion. The electrochemical impedance spectroscopy was utilized to investigate the charge transport characteristics of both modified and unmodified glassy carbon electrodes. A 5 mM aqueous solution of potassium ferrocyanide served as the redox probe, complemented by 0.1 M potassium chloride as the supporting electrolyte. The frequency was varied from 1 MHz to 1 Hz, with a consistent amplitude of 10 mV maintained throughout the measurements. Nyquist plots derived from the EIS data for GCE, TiO_2_/GCE, NH_2_-fMWCNTs/GCE, and NH_2_-fMWCNTs/TiO_2_/GCE are presented in Fig. 3(a). The EIS results indicate that the charge transfer resistance for NH_2_-fMWCNTs/TiO_2_/GCE is lower than that of all other modified and unmodified GCEs, suggesting reduced impedance during the charge transfer process. This low impedance significantly enhances sensor performance by facilitating greater sensitivity and quicker response times due to diminished resistance in the circuit. Consequently, this leads to an improved signal-to-noise ratio, providing clearer and more reliable readings, particularly at low analyte concentrations. Furthermore, low impedance reduces power consumption, which is beneficial for portable applications. Ultimately, sensors characterized by low impedance offer wider detection ranges and enhanced accuracy, making them suitable for precise, real-time monitoring.^29,30^ The effective fabrication of the electrode is validated by alterations in impedance parameters, which signify increased conductivity and expedited charge transport between the probe and the transducer. The impedance parameters derived from the model-fitted data are detailed in Table S1.† The electroactive surface area of the electrodes was examined through a cyclic voltammetric experiment conducted at room temperature in 5 mM K_3_[Fe(CN)_6_] and 0.1 M KCl. Fig. 3(b) shows the cyclic voltammetric behavior of the redox probe at the surface of GCE, TiO_2_/GCE, NH_2_-fMWCNTs/GCE, NH_2_-fMWCNTs/TiO_2_/GCE at scan rate of 100 mV s^−1^.

Another important component affecting the sensor performance is area of the electrode. The electroactive surface area of the bare and modified electrodes (Table S2†) was evaluated according to the Randles–Sevcik equation: *I*_pa_ = 2.6 × 10^5^*n*^3/2^*D*^1/2^*Cν*^1/2^*A*, applicable at 25 °C. In this equation, *I*_pa_ denotes the anodic peak current, *n* represents the number of electrons participating in the reaction, *ν* indicates the scan rate, *D* refers to the diffusion coefficient, *A* is the area of the electrode, and *C* signifies the concentration of the redox probe. All modified electrodes displayed improved current responses and enhanced reversibility compared to the bare GCE, with NH_2_-fMWCNTs/TiO_2_/GCE achieving the highest performance among them. The NH_2_-fMWCNTs/TiO_2_/GCE shows a fourfold increase in surface area relative to the unmodified GCE. A large electrode surface area greatly enhances sensor functionality by providing more active sites for analyte molecules. This enhancement enables analyte molecules to better align their electroactive components with the electrode, leading to stronger current signals that improve detection accuracy, even at low concentrations. Moreover, an increased surface area boosts the signal-to-noise ratio, thereby enhancing the precision and reliability of the sensor. Additionally, this larger area facilitates faster reaction kinetics and reduces response time, which increases the sensor's efficiency for real-time applications.^31^

To evaluate the performance of the designed modifiers towards the oxidation of BTB dye square wave voltammetry was carried out. The integration of NH_2_-fMWCNTs with TiO_2_ exhibited the greatest current sensitivity towards the target analyte, likely attributable to the modifier's elevated active surface area and reduced charge transfer resistance in comparison to GCE, TiO_2_/GCE, and NH_2_-fMWCNTs/GCE alone. The oxidation signal of the BTB dye at the unmodified electrode was detected at 0.55 V. Upon modification of the glassy carbon electrode with NH_2_-fMWCNTs and TiO_2_, there was no significant change in the oxidation potential of the analyte; however, the peak current intensity exhibited a fourfold increase, as demonstrated in Fig. 3(c).

### 3.3 Effect of scan rates

**Fig. 4:**
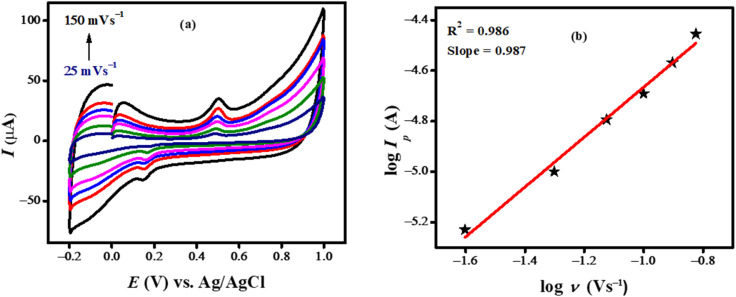
(a) Cyclic voltammograms showing the impact of the scan rate on the oxidative peak current intensity of 10 μM bromothymol blue in PBS electrolyte of pH 7.0; (b) plot of the log of peak current of bromothymol blue as a function of log of scan rate.

Cyclic voltammetry was used to examine the scan rate effect on the anodic peak current of BTB dye. The intensity of the peak current increased linearly with increase in scan rate and the peak potential slightly shifted towards greater potential with the increase as illustrated in Fig. 4(a). It can be seen from the CV signature that the reverse peak almost disappears at lower scan rate (25 mV s^−1^) however at higher scan rate both anodic and cathodic peaks appear in the positive potential domain. The kinetics of adsorption–desorption and surface-mediated processes are related to this behavior. At lower scan rate there is more time for desorption which inhibits the reduction of oxidized specie, which causes the reversal peak to vanish. Because of their extended residence time, the oxidized species may go through various chemical reactions that result in a product that is difficult to reduce which also contributes to the reduction peak's disappearance. The plot shown in Fig. 4(b) indicates adsorption regulated oxidation process as evident from its slope value of 0.987. The adsorptive nature of the process is validated by the correlation coefficient between the peak current and the square root of the scan rate (Fig. S1b†), which is lower than that between the peak current and the scan rate (Fig. S1a†).

### 3.4 Optimization of experimental settings

**Fig. 5:**
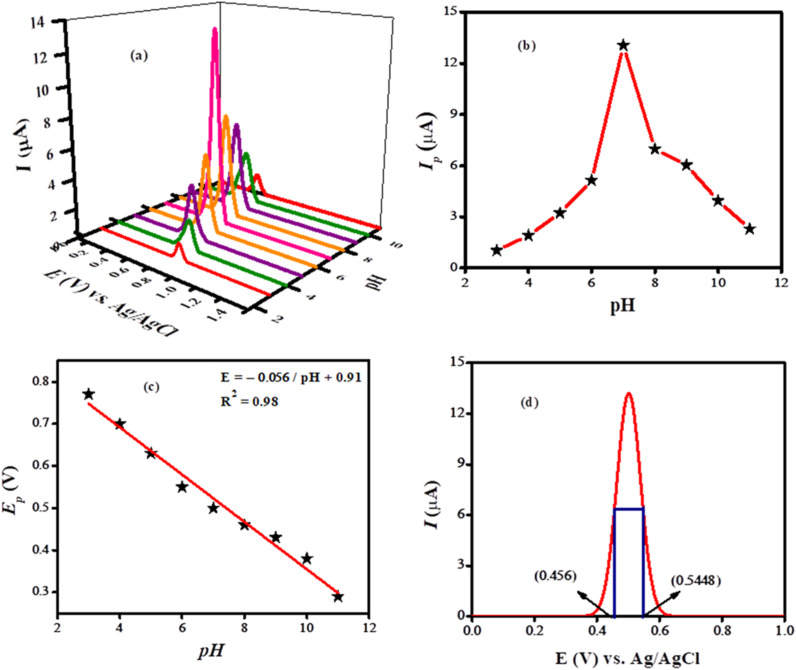
(a) SWVs of 10 μM bromothymol blue obtained by using NH_2_-fMWCNTs/TiO_2_/GCE in PBS solutions of different pH (3–11) at a scan rate of 100 mV s^−1^; (b) plot of *I*_p_ of bromothymol blue *vs.* pH of the media; (c) plot of *E*_p_ of bromothymol blue *vs.* pH of the solutions; (d) FWHM calculated from voltammogram recorded at the modified GCE in medium of pH 7.0.

A number of experimental parameters were optimized for getting the maximum peak current response of BTB. As supporting electrolyte can affect the intensity, potential and shape of the analyte signal, so SWV of the BTB was carried out at the modified electrode in various supporting electrolytes such as PBS (pH 6), BRB (pH 6) KOH, NaOH and KCl as illustrated in Fig. S2(a).† In comparison to other electrolytes, PBS was found more appropriate owing to the appearance of the intense and well-defined peak shape of the analyte in this medium. The designed sensor (NH_2_-fMWCNTs/TiO_2_/GCE) demonstrated the best response for BTB in PBS as evident from Fig. S2(b).† The voltammetric current response of BTB dye was investigated in PBS of different pH ranging from 3 to 11 as shown in Fig. 5(a). The maximum peak current was noticed in medium of pH of 7.0. The shift of peak potential with change in pH of the medium suggests proton participation during electron transfer reaction. The graph shown in Fig. 5(b) demonstrates proton coupled electron transfer reaction. The designed sensor is the most conductive for BTB at pH 7.0. Hence for the analytical detection of the dye PBS of pH 7.0 was chosen and rest of the experiments were conducted in this medium. The formula employed to calculate the ratio of protons to electrons in the redox process is expressed as Δ*E*_p_/ΔpH = 2.303 *mRT*/*nF*, with *m*/*n* representing the proton-to-electron ratio. The slope value derived from *E*_p_ – pH plot was 56 mV/pH which is closer to the Nernstian value of 59 mV/pH as seen in Fig. 5(c). This confirms that the oxidation reaction of BTB involves an equal number of protons and electrons. To confirm the exact number of electrons involved in the oxidation process the following formula was used: FWHM = 90 mV/*n*, where *n* represents the number of electrons and FWHM represents the full width at half maximum. The FWHM calculated from the voltammogram shown in Fig. 5(d) is 88.8 mV which is close to the theoretical value of 90 mV for the involvement of single electron transfer during electro-oxidation. Hence, the proton to electrons ratio (*m*/*n*) of 1 : 1 indicates one electron and one proton involvement in pH-dependent electro-oxidation.

BTB is a pH-sensitive dye characterized by a sulfone group and multiple conjugated aromatic rings. At a neutral pH of 7, it primarily exists in a deprotonated (anionic) form as a result of the proton loss from one of its phenolic hydroxyl group. The positively charged NH_2_-fMWCNTs enhance the adsorption of the dye through electrostatic interactions, while TiO_2_ nanoparticles contribute to the electrode's roughness, thereby increasing the availability of binding sites for analyte molecules. Together, these elements significantly enhance the sensitivity of the sensor. The oxidation process occurs in two stages: initially, an electron is transferred from the anionic dye molecule (BTB^−^) to the electrode, resulting in the formation of a neutral radical (BTB˙). Subsequently, a proton is transferred from the surrounding aqueous environment (PBS at pH 7), which stabilizes the radical and converts it into a neutral species.

The effect of deposition time on the peak current of BTB was probed in PBS of pH 7 using NH_2_-fMWCNTs/TiO_2_/GCE as depicted in Fig. S3(a).† The intense signal appeared in 5 s deposition time as evident from Fig. S3(b).† With further increase in the deposition time, the peak current intensity continuously decreases. Variation in deposition potential either towards more negative or more positive value influences the sensitivity of the developed sensors depending upon the nature of the analyte. The impact of deposition potential on the anodic peak current of the analyte was analyzed in PBS of pH 7.0. The deposition potential was varied from −0.2 to 0.3 V. The peak current of the BTB increased progressively with the elevation of deposition potential, as illustrated in the Fig. S3(c).† So, 0 V deposition potential was selected due to the maximum peak current response at this potential for further electrochemical investigations of the BTB dye. The higher signal intensity at a deposition potential of 0 V as shown in Fig. S3(d)† can be attributed to the fact that the targeted analyte had more access to the active sites on the modified electrode surface at this potential and the peak current decreases on further increase in deposition potential due to active sites saturation and multilayer accumulation that lowers the conductivity of the electrode.

### 3.5 Analytical performance of the designed sensor

#### 3.5.1 Limit of detection and calibration plot

**Fig. 6:**
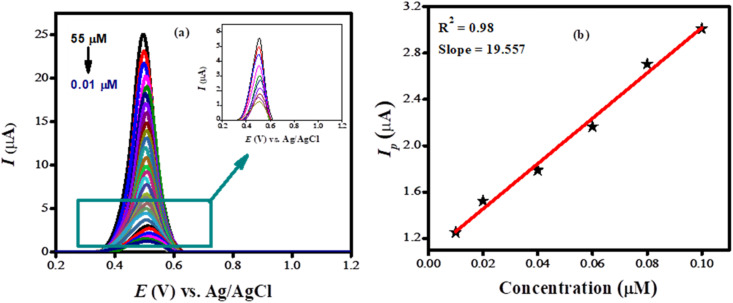
(a) SWVs of various concentrations (0.01–55 μM) of bromothymol blue obtained at NH_2_-fMWCNTs/TiO_2_/GCE in PBS of pH 7.0; (b) calibration plot of bromothymol blue.

SWV was performed under pre-optimized conditions to assess the detection capabilities of the sensing platform we developed. Fig. 6(a) shows the voltammograms obtained by varying concentrations of BTB dye ranging from 0.01 μM to 55 μM, demonstrating that the concentration of the targeted analyte directly influences the peak current. A linear calibration plot is depicted in Fig. 6(b). The limit of detection was calculated according to the formula: LOD = 3*σ*/*m*, where *m* signifies the slope and *σ* the standard deviation which was calculated from the peak current values of the blank solution. The limit of detection at the modified electrode (NH_2_-fMWCNTs/TiO_2_/GCE) was evaluated to be 0.1 nM, highlighting its potential as an effective tool for detection of the targeted analyte.

#### 3.5.2 Stability assessment of the designed sensor

The stability of the sensing scaffold was examined by monitoring the electrochemical response under pre-optimized experimental conditions. Stability was gauged through SWV at various time intervals, with the NH_2_-fMWCNTs/TiO_2_/GCE immersed in pH 7.0 PBS, as shown in Fig. S4(a).† Up to thirty hours, no significant change in the response of the electrode for the analyte was observed compared to the freshly modified electrode. These observations indicated intra- and inter-day stability of the sensor. The sustained peak current of BTB dye over time is attributed to the strong anchoring of the electrode modifier at the electrode surface. To assess the reliability of the designed sensor, four independent NH_2_-fMWCNTs/TiO_2_/GCEs were fabricated and SWV was subsequently performed under pre-optimized conditions. As illustrated in Fig. S4(b),† there were no significant changes in the signal intensity of the analyte, indicating that the electrochemical sensor demonstrated exceptional repeatability and amazing reproducibility.

**Fig. 7:**
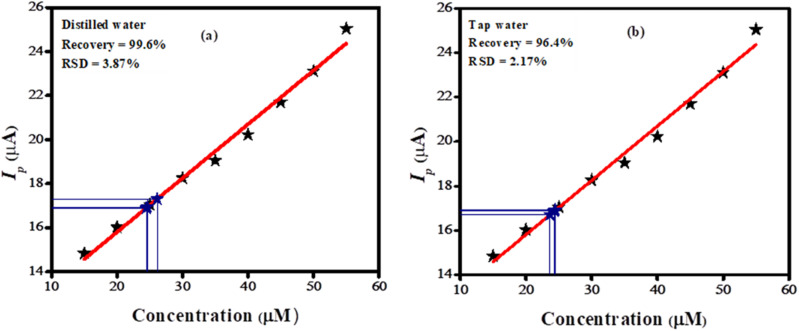
Calibration plot showing (a) recovery of 25 μM spiked amount of bromothymol blue in distilled water; (b) recovery of 25 μM spiked amount of bromothymol blue in tap water.

The BTB dye was dissolved in tap and distilled water to evaluate the accuracy and precision of the designed sensor. Initially, tests revealed no BTB molecules in either distilled or tap water. Following this, the practical applicability of the sensor was evaluated by spiking a known quantity of BTB using a standard addition procedure. A calibration plot was used to determine the recovered amount of BTB dye. Three replicates of each experiment were conducted, and the oxidation peak current was matched with the *I*_p_ displayed in the concentration profile of the linearity segment of the developed analytical method. The fabricated sensor demonstrated a high sensitivity towards BTB dye as validated by its relative standard deviation (RSD) of 2.17% in tap water and 3.87% in distilled water. The percentage recovery of the targeted analyte was found to be 99.6% in distilled water and 96.4% in tap water as shown in Fig. 7(a) and (b), highlighting the credible applicability of the designed sensor.

### 3.6 Photocatalytic degradation of BTB dye

**Fig. 8:**
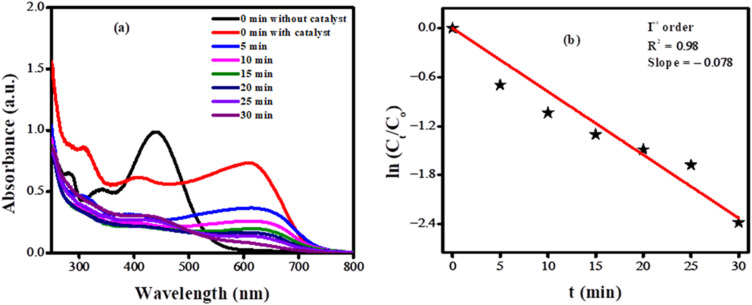
(a) UV-Vis spectra showing time based decrease in concentration of bromothymol blue dye due to photocatalytic breakdown by Ag_2_O NPs; (b) plot of 
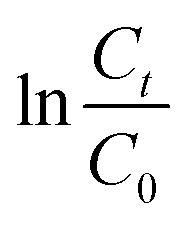
*vs. t* using UV-Vis spectroscopic data of the photocatalytic breakdown of bromothymol blue.

Ag_2_O nanoparticles were employed for the photocatalytic degradation of BTB when directly exposed to sunlight. UV-Vis spectroscopy was used to monitor the degradation. Catalyst dose, pH, and time were optimized to obtain the maximum degradation efficiency. The corresponding spectra under optimized conditions are shown in Fig. 8(a). To investigate the degradation rate, a number of kinetic models were used. The data fitted well in the pseudo-first-order kinetic model. The rate constant was evaluated from the expression: 
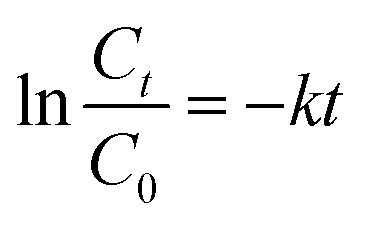
, where *C*_*t*_ indicates the absorbance at time “*t*”, *C*_0_ represents the absorbance before starting the experiment that is at “0” min. The rate constant “*k*” with a value of 0.078 min^−1^ was determined from the slope of the graph depicted in Fig. 8(b).

**Fig. 9:**
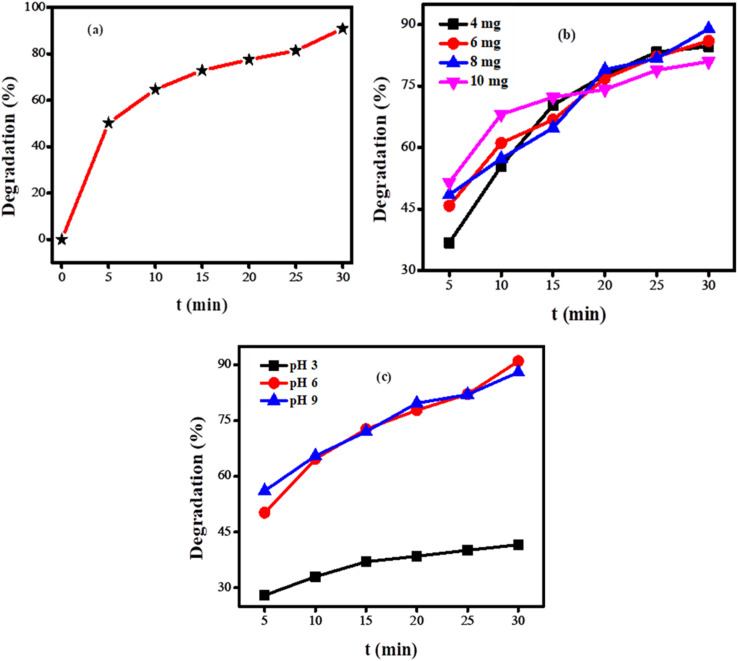
(a) Effect of irradiation time on the degradation of bromothymol blue dye; (b) effect of catalyst doze on the degradation of bromothymol blue; (c) effect of pH on the photocatalytic degradation of bromothymol blue.

Fig. 9(a) illustrates that initially, the rate of degradation is faster, which declines with time. This slowdown occurs because of the fewer active sites available for the dye molecules and the lower concentration of dye at the end of the reaction.^32^ The following relationship was used to determine the degradation efficiency: 
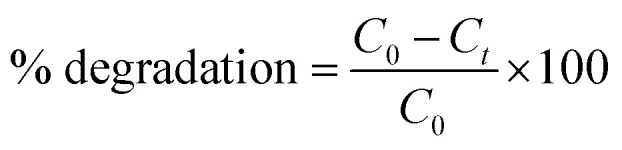
, where *C*_*t*_ indicates the absorbance at a specific time “*t*”, *C*_0_ represents the absorbance before exposure of sunlight. Ag_2_O degraded 92% of the BTB dye in 30 minutes under the exposure of light.

The amount of catalyst has both positive and negative impacts on the degradation of dyes. The number of photons absorbed increases with the increases of catalyst dose to some extent. A further enhancement in the loading of the catalyst decreases the efficiency of the photocatalyst by increasing the opacity of the solution.^32^ To investigate the impact of catalyst dosage on the percentage degradation of BTB dye, various catalyst amounts of 4 mg, 6 mg, 8 mg, and 10 mg were employed. The photocatalytic degradation of the dye was monitored for an equal interval of time. It was observed that 8 mg catalyst dose exhibited the highest catalytic efficiency as obvious from Fig. 9(b). Therefore, this catalyst dose was selected for further investigations of the photocatalytic breakdown of the dye.

Understanding the pH effect on the photocatalytic degradation of dyes is complex because degradation can take place through different mechanisms such as attack of hydroxyl radicals, reduction of dye by electrons present in the conduction band, and oxidation due to the holes present in the valence band. The electrostatic interaction between the dye and the catalyst is affected by the change in pH of wastewater. To find out how pH affects the percentage degradation of BTB dye, the degradation experiment was carried out in three different setups of pH 3, 6, and 9. It was observed that the degradation efficiency of BTB dye is higher at pH 6 as illustrated in Fig. 9(c). This pH facilitates the optimal adsorption between the analyte and the photocatalyst, which is crucial for the degradation. Furthermore, an acidic pH induces a positive charge on the surface of the photocatalyst, which enhances the likelihood of achieving maximum degradation efficiency for anionic dyes.

Table 1 shows a brief comparison of the photocatalytic degradation of BTB dye with the reported literature. However, Table 2 presents a concise comparison of Ag_2_O nanoparticles employed as photocatalysts for the degradation of various dye types, emphasizing their potential effectiveness in reducing dye contamination. Our synthesized catalyst demonstrated significant potential for the elimination of BTB dye, achieving a degradation rate of 92% within a 30 minutes timeframe.

**Table 1:** Comparison of the performance of Ag_2_O NPs with other catalysts used for the photocatalytic degradation of BTB dye

S. no.	Materials	Degradation efficiency	Time	pH	Ref.
1	MgFe_2_O_4_	65%	180 min	3	33
2	Ru–TiO_2_	95%	100 min	4	34
3	Ce–ZnO-rGo	92%	180 min	—	35
4	La-doped ZnO-rGO	81%	210 min	—	36
5	Ag_2_O NPs	92%	30 min	6	This work

**Table 2:** Comparsion of performance of Ag_2_O NPs as a photocatalyst for different types of dyes

S. no.	Pollutant name	Time (min)	Degradation efficiency (%)	Ref.
1	Acid orange 8	150	70	37
2	Methylene blue	180	97.7	38
3	Methylene orange	160	100	39
4	Rhodamine 6G	330	98.5	40
5	Bromothymol blue	30	92	This work

#### 3.6.1 Discoloration and mechanism of BTB dye

**Fig. 10:**
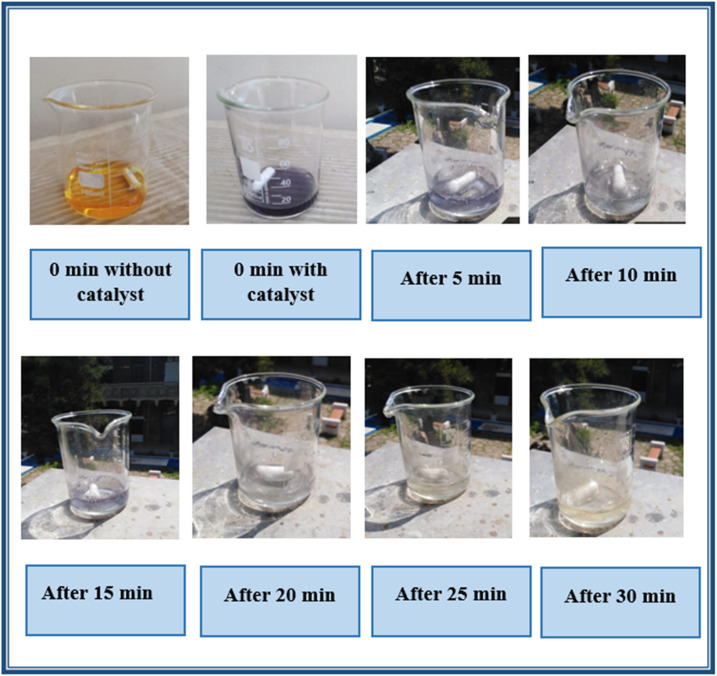
Photocatalytic degradation of bromothymol blue dye under direct sunlight.

To examine the effect on the discoloration of BTB dye solution, the change in color of the dye was observed by exposing 55 μM dye solution to sunlight under optimized conditions. Fig. 10 shows that the dye changes color from yellow to grey immediately after addition of the photocatalyst, which corresponds to a change in peak position from 434 nm to 618 nm which is attributed to the formation of charge-transfer complex which alters the electronic structure of the dye.BTB (yellow) + Ag_2_O NPs → Ag–BTB complex (grey)

Afterward, the color fades away with the passage of time which indicates the degradation of dye, which is also confirmed by the decrease in absorption intensity. This happens due to the irradiation of light on the photocatalyst creating electrons and holes, which in turn form hydroxyl radicals. The disintegration of the bromothymol blue complex occurs as a result of the attack by OH radicals, which possess the second highest oxidation potential.Ag_2_O + *hν* → Ag_2_O (e^−^ + h^+^)h^+^ + H_2_O → ·OH + H^+^e^−^ + O_2_ → O_2_^−^ → H_2_O_2_ → ·OH + OH^−^

### 3.7 Adsorptive removal of bromothymol blue from wastewater

**Fig. 11:**
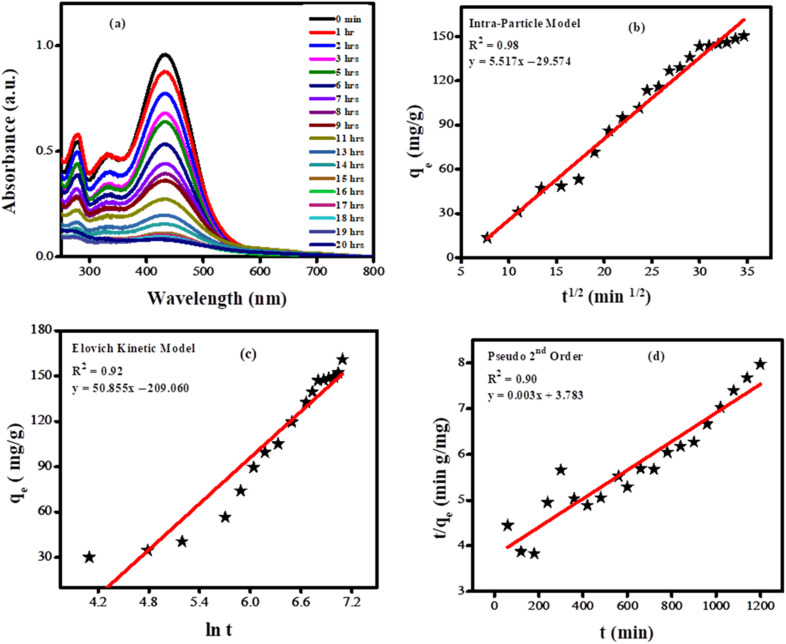
(a) UV-Vis spectra showing adsorption of bromothymol blue dye over Ni doped SnO_2_ NPs; (b) plot according to intraparticle kinetic model; (c) Elovich kinetic model; (d) pseudo second order kinetic model.

Numerous materials, including carbon nanotubes, graphene oxide, metal oxide nanoparticles, powdered leaves, ashes, and fruit peels, were evaluated for the adsorptive removal of BTB dye, with SnO_2_ nanoparticles demonstrating the highest adsorption efficiency. To expedite the process and enhance adsorption capacity, nickel was incorporated as a dopant in SnO_2_ nanoparticles, leading to successful adsorption of BTB dye using 4% Ni-doped SnO_2_ nanoparticles. The UV-Vis spectroscopic technique was utilized to track the adsorptive removal of the dye, with a calibration curve established beforehand to relate absorbance to concentration. The known concentration of the dye solution underwent UV-Vis spectroscopic analysis, and the calibration plot illustrating the relationship between absorbance and concentration is presented in Fig. S5.† The maximum dye removal efficiency was determined according to the formula: 
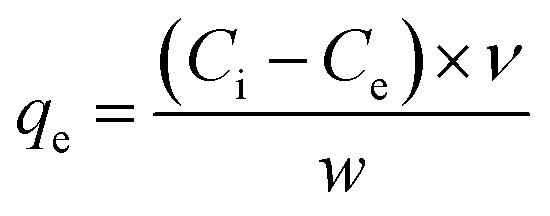
. The adsorption of BTB dye utilizing 4% Ni-doped SnO_2_ nanoparticles was assessed through UV-Vis spectroscopy under optimized conditions, which included an adsorbent concentration of 4 mg, a dye volume of 20 mL, a solution pH of 3, and contact duration of 20 hours. The corresponding UV-Vis spectra are presented in Fig. 11.

The adsorption data, acquired through UV-Vis spectroscopy, were analyzed using these three kinetic models using the intraparticle diffusion model, the Elovich kinetic model, and pseudo-second-order kinetics. The intra-particle diffusion is expressed by the equation: *q*_e_ = *k*_pi_*t*^1/2^ + *C*_i_, where, *q*_e_ is on the vertical axis, *t*^1/2^ on the horizontal axis, *k*_pi_ is the slope having units (mg g^−1^ min^−0.5^) and *C*_i_ is the intercept indicating the thickness of border layer, and *q*_e_ is the adsorption capacity at a specific time.^41^ In the Elovich kinetic model: 
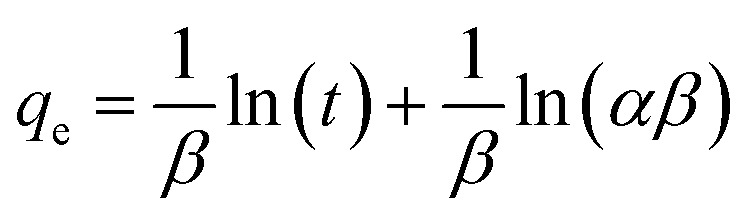
, *q*_e_ represents the adsorption capacity at a given time in mg g^−1^, *β* the adsorption constant (mg g^−1^ min^−1^) and *α* the initial adsorption rate expressed in mg g^−1^ min^−1^. This model explains how strongly the adsorbate absorbs over the surface of the adsorbent.^42^ By applying the pseudo-second-order kinetic model: 
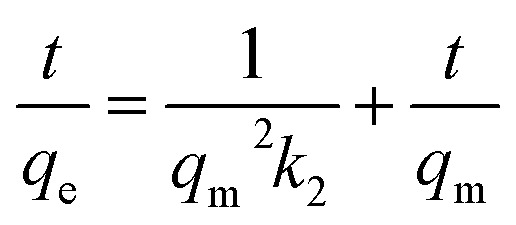
, the maximum adsorption capacity (*q*_m_) and the rate constant (*k*_2_) are obtained from the slope and intercept of the graph between 
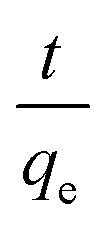
 and *t*.^43^

The intraparticle kinetic model showed better correlation coefficient as shown in Fig. 11(b). Intraparticle kinetic model showed better correlation coefficient (*R*^2^). The analysis of the plot indicates that the trend line does not intersect the origin, thereby supporting the conclusion that the adsorption process is governed by surface diffusion, which is the rate-limiting step. The intraparticle diffusion rate constant for the adsorption of bromothymol blue was evaluated to be 5.517 mg g^−1^ min^−1/2^.

#### 3.7.1 Adsorption isotherms

The adsorption data were analyzed using the Temkin, Freundlich, and Langmuir isotherms. These isotherms provide insights into the uniformity or variability of the adsorbent's surface and offered information regarding the possible interactions between the adsorbent and the adsorbate. The linear forms of Temkin, Freundlich, and Langmuir adsorption equations are given as: *q*_e_ = *B* ln *A* + *B* ln *C*_e_, 
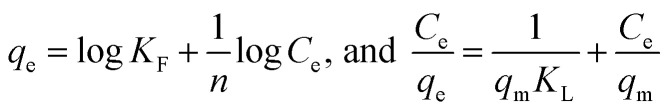
 respectively. Where *q*_m_ and *q*_e_ denote the maximum and time-dependent adsorption capacities, *C*_e_ stands for the equilibrium concentration of bromothymol blue, *B* in the Temkin equation represents the heat of adsorption (*B* = *RT*/*b*), Langmuir constant (*K*_L_) and Freundlich constants (*K*_F_).

**Fig. 12:**
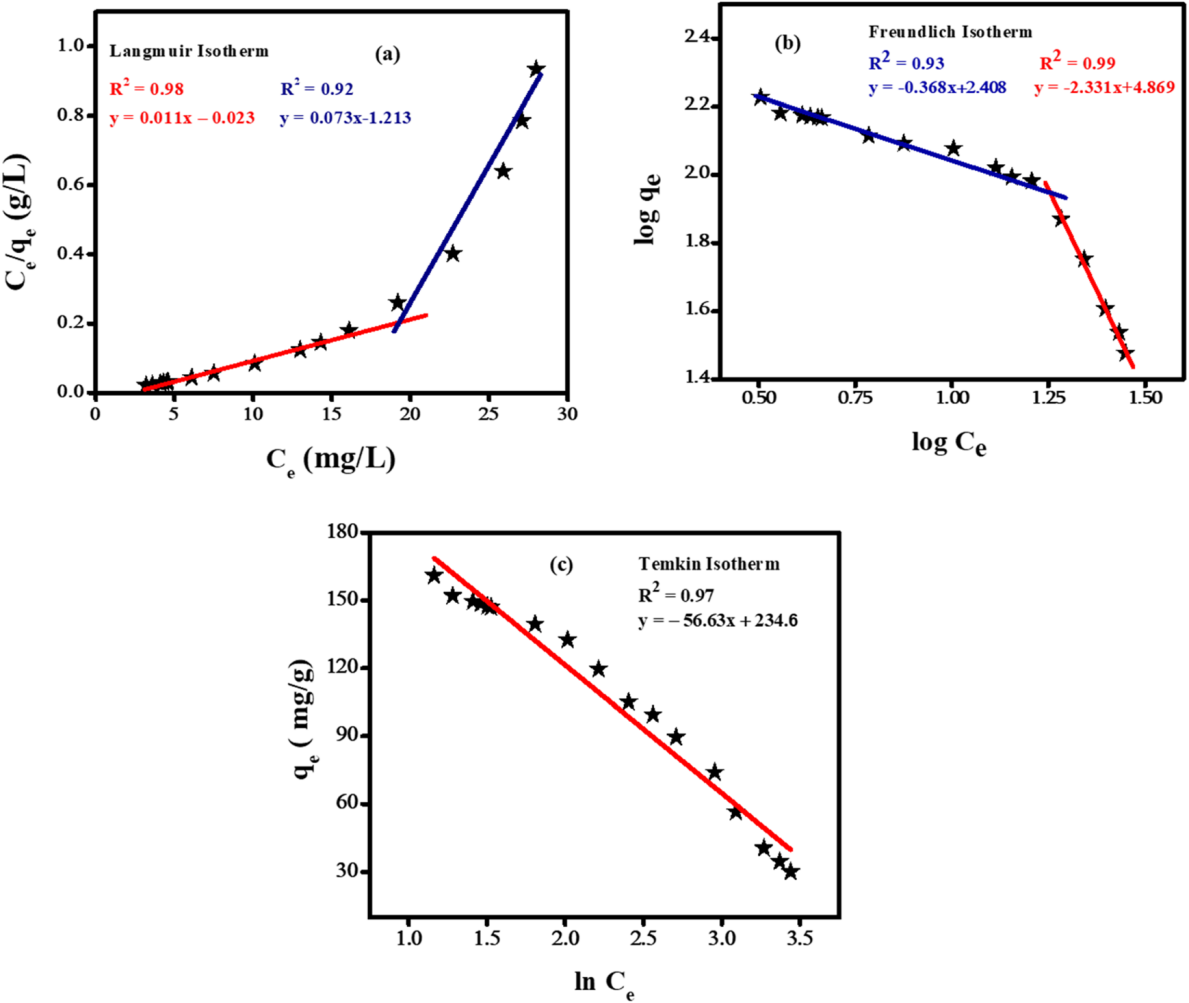
Fitting of adsorption data in (a) Langmuir isotherm (b) Freundlich isotherm (c) Temkin isotherm.

Fig. 12 illustrates the different isotherms utilized to obtain various parameters for the adsorptive removal of BTB dye. The separation factor (*R*_L_), referred to as the dimensionless Langmuir constant, is employed to elucidate the fundamental characteristics of adsorption. The *R*_L_ value determines the nature of adsorption: 
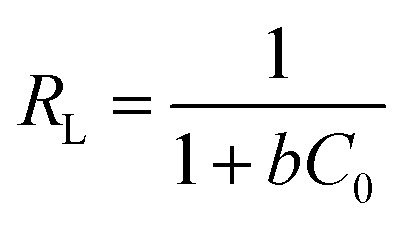
. A zero value of *R*_L_ indicates an irreversible adsorption process, 1 represents a favorable adsorption and greater than 1 denotes an unfavorable adsorption process.^44^

The distinctive adsorptive behavior of bromothymol blue dye on Ni-doped SnO_2_ NPs is explained by the presence of adsorption sites and the changing surface contacts. The predominance of the Langmuir isotherm at lower dye concentrations is consistent with the monolayer development. On the other hand, multilayered adsorption at higher concentration occurs because of surface site saturation as indicated by the dominance of the Freundlich pattern of adsorption at higher concentration. The variation of adsorbate–adsorbent interactions at different concentrations is highlighted by this switching behavior. The intricate relationship between surface chemistry and adsorption energetics was further validated by the Temkin isotherm, which takes into account variations in the heat of the adsorption process and consistently aligns with the observed data. Table 3 lists the corresponding parameters that were obtained from various isotherms.

**Table 3:** List of adsorption parameters of bromothymol blue obtained from various isotherms

Isotherm	Parameters	Values
Langmuir	*q*_m_	90.90 mg g^−1^
	*K*_L_	0.48 L mg^−1^
	*R*_L_	0.06
Freundlich	*N*	0.429
	*K*_F_	6.3 × 10^4^ mg g^−1^
Temkin	*B*	56.63 mg g^−1^
	*A*	62.80 L g^−1^

Literature survey indicates that tin oxide-based nanoparticles possess considerable promise as adsorbents for a range of pollutants. A. Nilchi and coworkers discovered that nano tin oxide reached maximum adsorption capacities of 66.6 mg g^−1^ for uranium and 62 mg g^−1^ for thorium under optimized conditions.^45^ S. Davoodi *et al.* developed tin oxide nanoparticles supported on activated carbon (SnO_2_-NP-AC), which exhibited a maximum adsorption capacity of 67 mg g^−1^ for murexide dye.^46^ Wajid Rehman and colleagues created a binary nanocomposite incorporating tin and titanium oxide, which served as an effective adsorbent for lead ions, demonstrating a *q*_max_ of 70 mg g^−1^.^47^ Our work introduces Ni-doped SnO_2_ nanoparticles for bromothymol blue, achieving a maximum adsorption capacity of 91 mg g^−1^, that highlights the versatility of tin oxide in removing pollutants from wastewater.

## 4 Conclusions

Nanostructured TiO_2_, Ag_2_O, and Ni-doped SnO_2_ nanoparticles were synthesized using environmentally friendly methods and proved effective as electrode modifiers, photocatalysts, and adsorbents for the detection and removal of bromothymol blue (BTB) dye from wastewater. The incorporation of TiO_2_ nanoparticles and amino-functionalized multi-wall carbon nanotubes (NH_2_-fMWCNTs) significantly enhanced the peak current response of bromothymol blue, enabling the NH_2_-fMWCNTs/TiO_2_/GCE sensor to detect BTB concentrations as low as 0.1 nM under optimized conditions (PBS supporting electrolyte, pH 7, deposition time of 5 s and deposition potential of 0 V). Degradation experiments revealed that Ag_2_O nanoparticles can photocatalytically degrade BTB dye up to 92% in just 30 minutes. The results from the adsorption experiments indicated that Ni-doped SnO_2_ possesses a significant adsorption capacity of 91 mg g^−1^, aligning with the Langmuir–Freundlich isotherm model. Kinetic analyses showed that the degradation followed a pseudo-first-order mechanism, accompanied by intraparticle adsorption processes.

## Data availability

The authors declare that the data are available in this manuscript in the form of tables and figures.

## Conflicts of interest

The authors declare no conflict of interest regarding the publication of this manuscript.

## Supplementary Material

RA-015-D4RA08296F-s001
