# Supplementary material for: Nanosensor for the detection of bromothymol blue dye and its removal from wastewater by sustainable methods
Source: RSC Adv. 2025 Feb 7;15(6):4203–19. doi: 10.1039/d4ra08296f (PMC11803445; doi:10.1039/d4ra08296f)
Supplement: RA-015-D4RA08296F-s001 [file RA-015-D4RA08296F-s001.pdf]

Supplementary Information (SI) for RSC Advances.
This journal is © The Royal Society of Chemistry 2025

Nanosensor for the Detection of Bromothymol Blue Dye and its Removal from Wastewater by Sustainable Methods

Nashra Sheraz[a], Afzal Shah[a]*, Syed Sakhawat Shah[a]

[a]Department of Chemistry Quaid-i-Azam University, Islamabad 45320, Pakistan

*Correspondence: afzals_qau@yahoo.com

Table S1: Equivalent circuit model-fitted to experimental impedance data for the evaluation of EIS parameters through modified and unmodified GCEs.

**Working electrode**	**R_S (Ω)**	**R_{ct} (Ω)**
Bare GCE	99.06	4214
TiO_2/GCE	102.1	3067
NH_2-*f*MWCNTs/GCE	104.6	1562
NH_2-*f*MWCNTs/TiO_2/GCE	108.5	515.2

Table S2: Surface area of bare and modified electrodes calculated according to Randles-Sevcik equation.

**Working electrode**	**Surface area (cm^2)**	**Peak separation (mV)**
Bare GCE	0.027	87
TiO_2/GCE	0.048	82
NH_2-*f*MWCNTs/GCE	0.068	78
NH_2-*f*MWCNTs/TiO_2/GCE	0.085	72

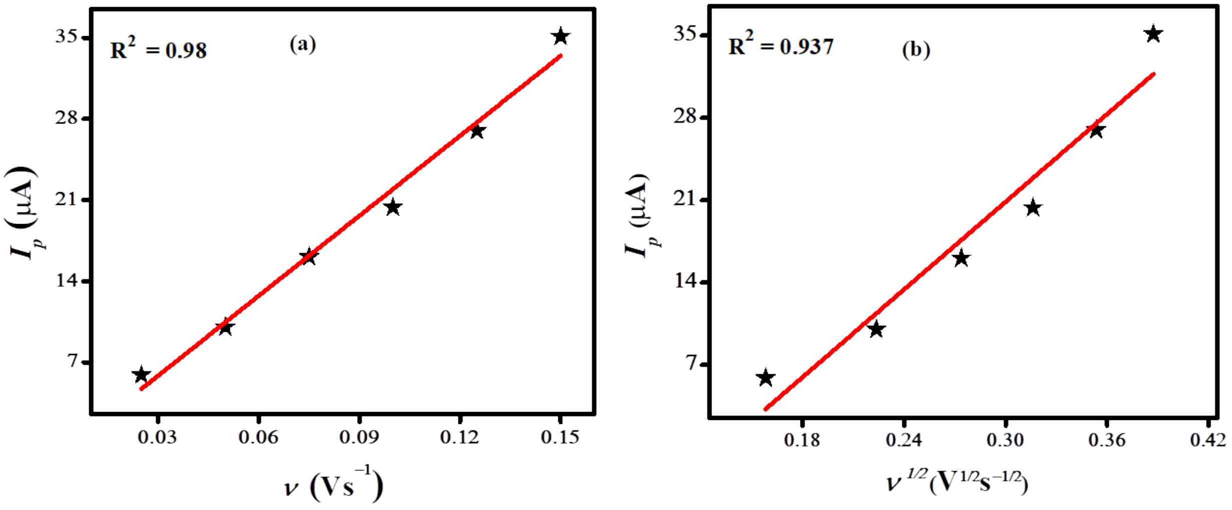

Figure S1: (a) Plot of peak current vs. scan rate; (b) Plot of peak current vs. square root of scan rate.

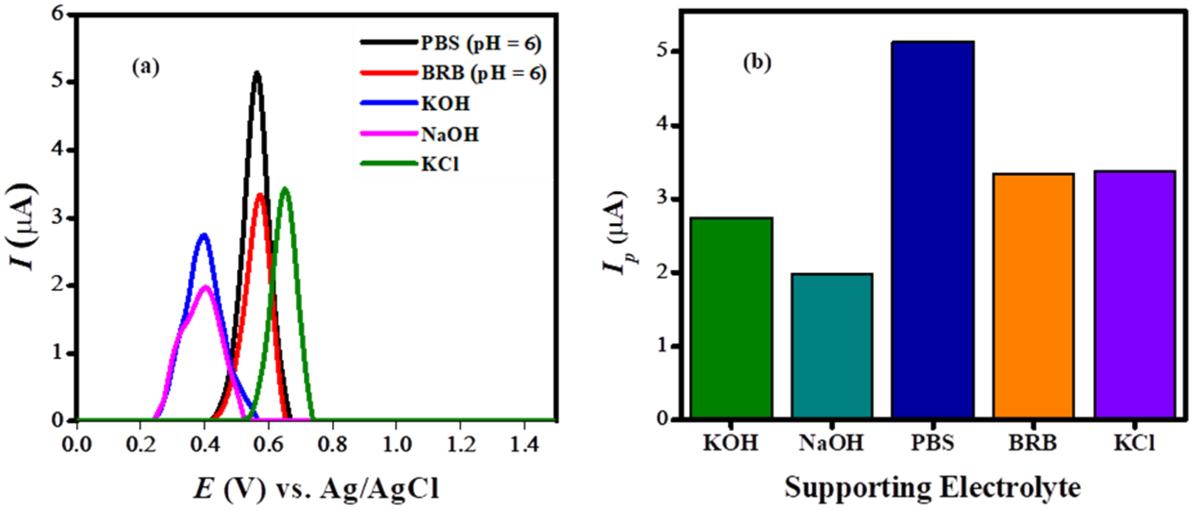

Figure S2: (a) Impact of supporting electrolytes on the anodic peak current of 10 μM bromothymol blue using NH_2-*f*MWCNTs/TiO_2 modified GCE; (b) Bar graph showing maximum peak current of bromothymol blue in different supporting electrolytes.

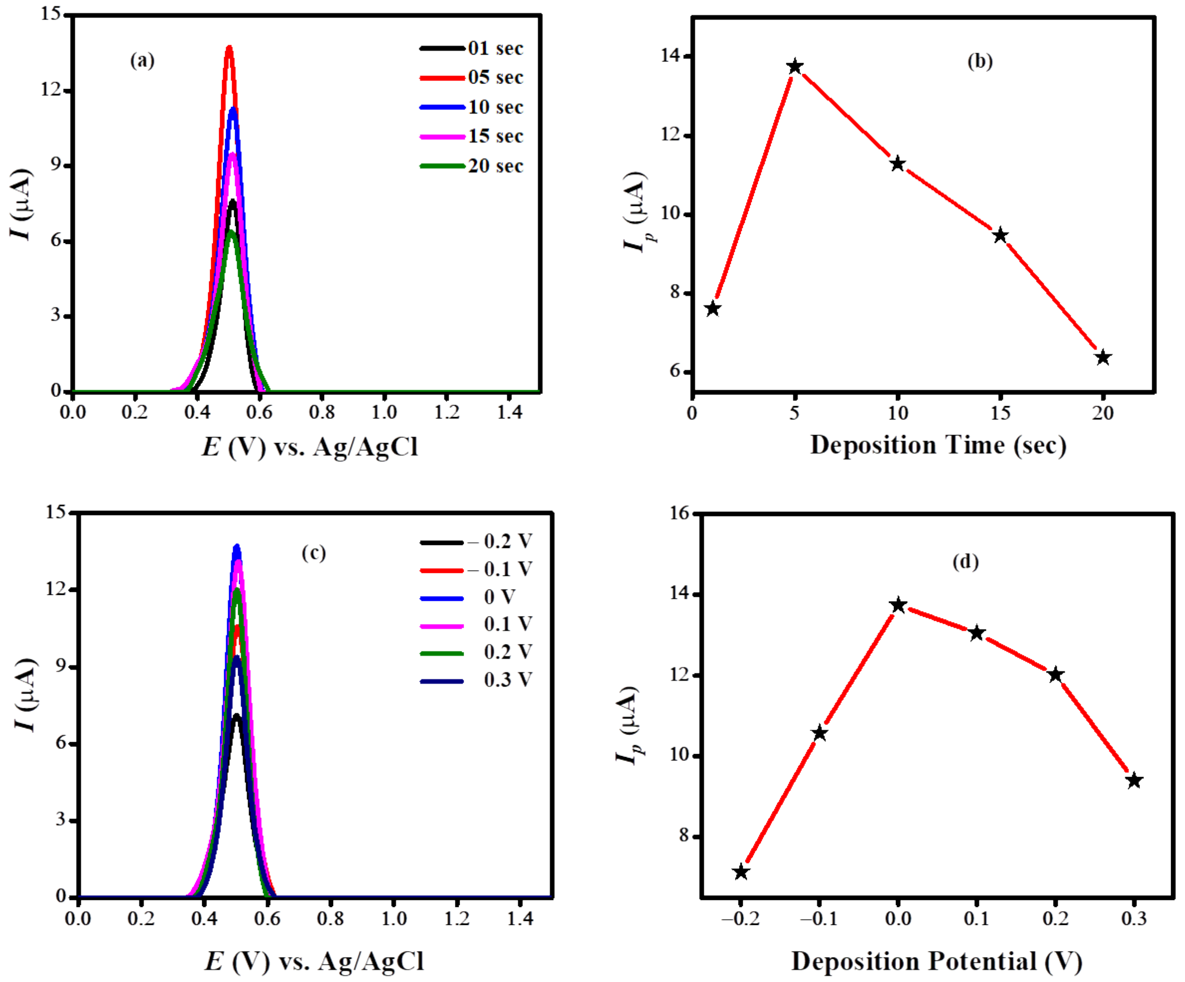

Figure S3: (a) Impact of deposition time on the peak current of 10 μM bromothymol blue obtained at NH_2-*f*MWCNTs/TiO_2/GCE in PBS of pH 7.0 at a deposition potential of 0 V;(b) Plot of I_p of bromothymol blue vs. deposition time; (c) Impact of deposition potential on the peak current intensity of 10 μM bromothymol blue in PBS having pH 7.0; (d) Plot of I_p of bromothymol blue vs. deposition potential.

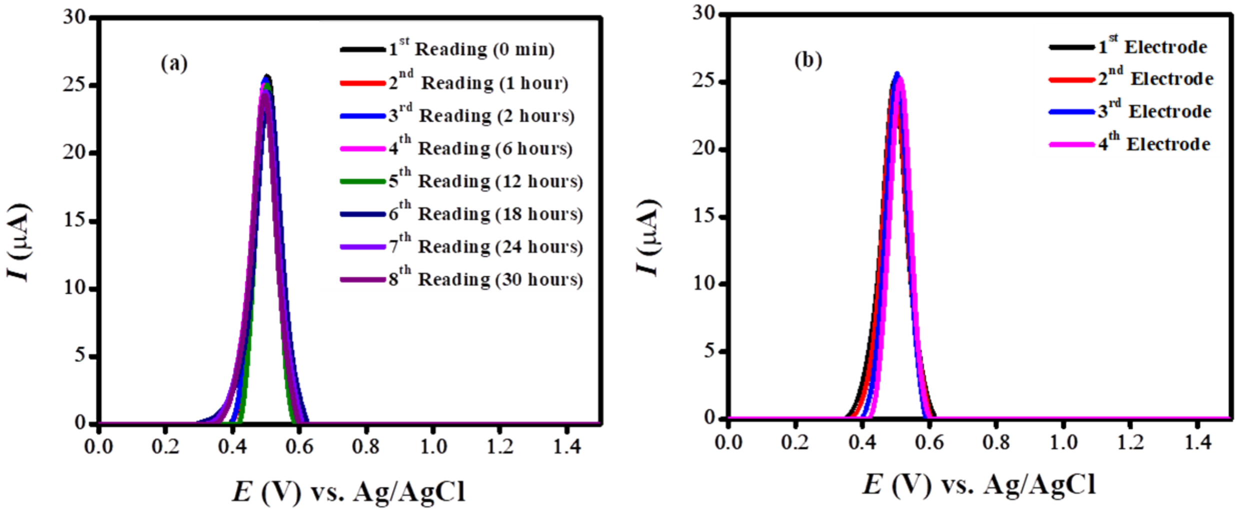

Figure S4: (a) SWVs of bromothymol blue using modified GCE at different time intervals showing repeatability of the designed sensor; (b) SWVs of bromothymol blue using different modified GCEs in phosphate buffer saline of pH 7.0.

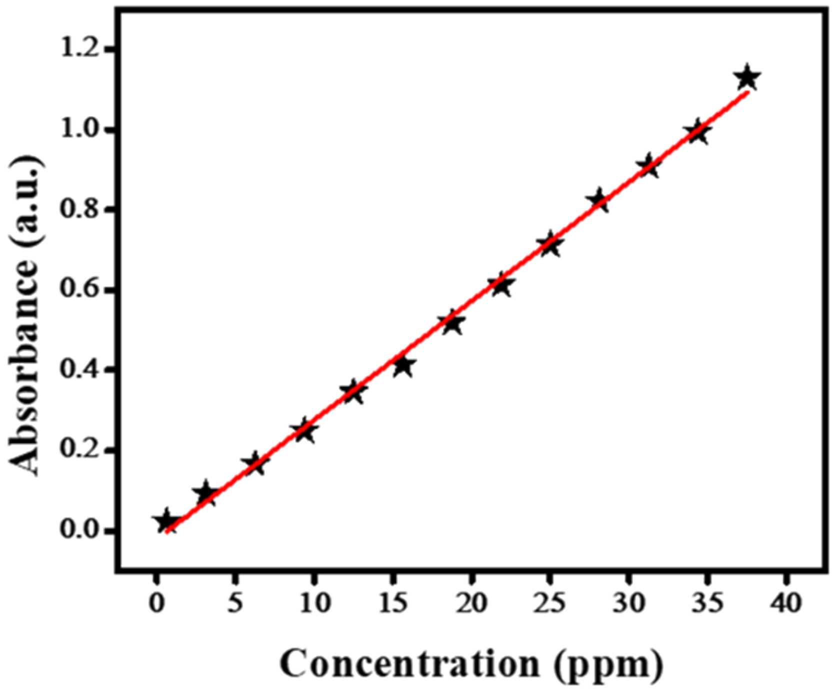

Figure S5: Calibration curve between concentration of bromothymol blue dye and corresponding absorbance.
